# Single-Sampling Strategy vs. Multi-Sampling Strategy for Blood Cultures in Sepsis: A Prospective Non-inferiority Study

**DOI:** 10.3389/fmicb.2020.01639

**Published:** 2020-07-23

**Authors:** David Yu, Anna Larsson, Åsa Parke, Christian Unge, Claes Henning, Jonas Sundén-Cullberg, Anna Somell, Kristoffer Strålin, Volkan Özenci

**Affiliations:** ^1^Division of Clinical Microbiology, Department of Laboratory Medicine, Karolinska Institutet, Stockholm, Sweden; ^2^Functional Area of Emergency Medicine, Karolinska University Hospital, Stockholm, Sweden; ^3^Department of Medicine Huddinge, Karolinska Institutet, Stockholm, Sweden; ^4^Department of Infectious Diseases, Karolinska University Hospital, Stockholm, Sweden; ^5^Clinical Microbiology Laboratory, Södra Älvsborg Hospital, Borås, Sweden; ^6^Functional Area of Perioperative Medicine and Intensive Care, Karolinska University Hospital, Stockholm, Sweden; ^7^Department of Clinical Microbiology, Karolinska University Hospital, Stockholm, Sweden

**Keywords:** bloodstream infection, blood culture, sampling, sepsis, bacteria, contamination

## Abstract

**Background:**

Optimal sampling is critical for the performance of blood cultures (BCs). Most guidelines recommend collecting 40 ml of blood, divided between two venipuncture sites, i.e., multi-sampling strategy (MSS). Sampling through a single venipuncture site, i.e., single-sampling strategy (SSS) is easier; however, the diagnostic performance of SSS compared to MSS remains unknown. Thus, we aimed to study if SSS is non-inferior to MSS for detection of pathogenic microorganisms.

**Methods:**

A prospective, paired, non-inferiority design was used. Patients with clinically suspected sepsis admitted to an Emergency Department were included. Six BC bottles were simultaneously collected, consisting of four BC bottles from the first arm and two from the other arm. SSS consisted of BC bottles 1, 2, 3, and 4, and MSS consisted of BC bottles 1, 2, 5, and 6. Samples were incubated in a BacT/ALERT BC system.

**Results:**

The final analysis included 549 episodes. Pathogenic microorganisms were detected in 162 cases (29.5%) with MSS and 160 cases (29.1%) with SSS, yielding an absolute difference of 0.36%, with a 95% confidence interval of -1.33 to 2.06%, which did not exceed the predefined non-inferiority margin of 5%. MSS tended to produce more contaminant growth (7.3% of cases) than SSS (5.3% of cases; *p* = 0.072).

**Conclusion:**

The study showed that SSS was non-inferior to MSS in detecting pathogenic microorganisms and supports the use of SSS as a routine method.

## Introduction

Sepsis is defined as a life-threatening organ dysfunction caused by a dysregulated host response to infection (Singer et al., 2016; Seymour et al., 2017). Early optimal blood culture (BC) sampling followed by initiation of antibiotic therapy is a cornerstone of sepsis management (Rello et al., 2017; Levy et al., 2018), and BC is widely accepted as the gold standard for microbiological diagnostics in sepsis (Lamy et al., 2016; Rello et al., 2017).

The goal of BC sampling should be to collect a sufficient volume of blood to optimize the chance to detect pathogenic microorganisms and minimize sample contamination risk. The diagnostic yield of BC is strongly correlated to the volume of blood collected and cultured (Cockerill et al., 2004; Patel et al., 2011; Banerjee et al., 2016; Henning et al., 2019). Currently, a total of 32–40 ml blood is recommended for BC to achieve relevant detection rates (Cockerill et al., 2004; Lee et al., 2007; Patel et al., 2011). Most guidelines recommend at least two sets of BCs, each consisting of one aerobic and one anaerobic bottle (Patel et al., 2011; Lee et al., 2007; Patel et al., 2011). However, recommendations are scarce regarding the optimal number of bottles and venipunctures also referred to as sampling strategy. This includes recent international guidelines for sepsis, which do not specify a recommended sampling strategy (Rhodes et al., 2017).

Two main sampling strategies are in current clinical use, i.e., the well-established multi-sampling strategy (MSS), with sampling from two venipuncture sites, and the newer single-sampling strategy (SSS), with the whole desired volume of blood collected from one venipuncture site. Because of collection from two sites, MSS has been claimed to allow for better discrimination between true bacteremia and contamination than SSS (Lamy et al., 2016). However, the two venipunctures of MSS may result in a higher risk of contamination (Dargere et al., 2014). In addition, it is reasonable to assume that MSS may also result in the collection of only two BC bottles through one venipuncture due to heavy workload or patient-related factors. In contrast, SSS theoretically implies less contamination rates because of fewer venipunctures and a higher rate of four BC bottles sampled (Lamy et al., 2016). The major difference between SSS and MSS in clinical practice is that SSS is related to reduced patient discomfort and less labor-intensive sampling. Currently, MSS is the predominant approach in most countries, but SSS is gaining approval as a safe alternative (Lamy et al., 2016).

Hitherto published studies on comparison of BC sampling strategies are scarce and include heterogeneous patient groups undergoing BC sampling. A recent comparative study including 245 patients with signs of infection suggested that SSS may be superior to MSS by a composite outcome defined as detection rate of both clinically relevant growth and contamination (Dargere et al., 2014). However, in this study, the second venipuncture was not taken simultaneously but allowed to occur up to 24 h after the first venipuncture which may have affected the conditions under which the second sampling was done, such as concentration of pathogens in blood, administration of antibiotic therapy, and the clinical condition of the patient.

The few and diverging guidelines concerning BC sampling strategies highlight a lack of evidence to support a consensus recommendation. Therefore, there is an utmost need for clinical data comparing the performance of SSS and MSS in patients with sepsis.

The aim of the present study was to assess non-inferiority of SSS vs. MSS in a well-characterized patient group with clinically suspected sepsis. The primary outcome was the difference between detection rates of pathogenic microorganisms in SSS and MSS. The secondary outcome measure was contamination rates of SSS and MSS.

## Materials and Methods

### Study Design

This prospective non-inferiority study involved a paired design for comparing SSS and MSS. The primary outcome measure was detection of pathogenic microorganisms in BC. The study was performed at Karolinska University Hospital Huddinge, Stockholm, Sweden, a tertiary care hospital with 700 beds.

In the hospital’s Emergency Department, all patients are routinely subjected to triage with the Rapid Emergency Triage and Treatment System (Ljunggren et al., 2016), and a sepsis alert is triggered for triage signs of organ dysfunction combined with signs of infection, i.e., fever, history of fever, or clinical suspicion of infection. The sepsis alert’s trigger signs of organ dysfunction are either A or B, i.e., A) at least one of oxygen saturation below 90% despite supplemental oxygen administration, respiratory rate greater than 30 per min, heart rate greater than 130 beats per min, systolic blood pressure under 90 mmHg, or Glasgow Coma Scale below 8; or B) blood lactate greater than 3.2 mmol/L combined with at least one of oxygen saturation below 95% on room air, respiratory rate greater than 25 per min, heart rate greater than 110 beats per min, altered mental status, and temperature above 38.5°C or below 35°C. Patients who trigger the sepsis alert are subjected to urgent multidisciplinary bedside assessment by physicians from the Emergency Department, Department of Infectious Diseases, and Intensive Care Unit to optimize clinical assessment and treatment.

Three BC sets, each one consisting of an aerobic (BactAlert FA Plus) and an anaerobic (BactAlert FN Plus) BC bottle, were sampled from each patient who triggered the sepsis alert system. The BC bottles were labeled with numbers 1 through 6. MSS consisted of bottles 1, 2, 3, and 4, and SSS consisted of bottles 1, 2, 5, and 6. Bottles 1 through 4 were sampled from the same venipuncture site. Bottles 5 and 6 were collected from the other site. Bottles 1, 3, and 5 were BactAlert FA Plus bottles, and bottles 2, 4, and 6 were BactAlert FN Plus bottles. BC bottles were sampled consecutively in the labeled order. In cases of indwelling intravascular catheters, bottles 1–4 were sampled from the catheter. When comparing MSS to SSS, microbiological data from all six BC bottles were used as reference.

### Inclusion and Exclusion Criteria

Consecutive adult patients who triggered the sepsis alert from September 2017 to February 2019 were included in the study. A suspected sepsis episode, here referenced as an “episode,” was defined as a patient who triggered the sepsis alert system. Two or more episodes from the same patient could be included if they occurred more than 72 h apart. Episodes were excluded if fewer than six BC bottles were collected or if collection of blood was not performed in accordance with the study protocol.

### Microbiology Procedures

The BC bottles were transported to the Department of Clinical Microbiology, Karolinska University Hospital, according to routine practice and were analyzed according to the standard routine of that department (laboratory). The labeling specific for the present study was documented in the laboratory data system. From the beginning of the study in September 2017 until September 20, 2018, the BC bottles were incubated in BacT/ALERT 3D (Bio-Merieux, France). Beginning on September 20, 2018, the clinical microbiology laboratory changed its BC system to BacT/ALERT Virtuo (Bio-Merieux, France). The BactAlert FA Plus and BactAlert FN Plus BC bottles were used throughout the study. BCs were cultured until they signaled positive or for a total of 5 days. In positive BCs, samples were Gram stained and then subcultured on agar plates. Colonies that grew on agar were subjected to species identification by matrix-assisted laser desorption/ionization time-of-flight mass spectrometry (Bruker Daltonik, Bremen, Germany) and were subjected to antibiotic susceptibility testing by standard laboratory procedures.

### Relevant Growth and Contaminant Growth

Information regarding BC results in terms of isolate identification and time to detection was collected from the laboratory information system. Detected isolates were defined as clinically relevant growth or contaminant growth, according to an improved version of the methods used in previously published reports (Bekeris et al., 2005; Dawson, 2014). Relevant growth was defined as growth of pathogenic microorganisms in at least one BC bottle.

Contaminated episodes were defined as such if two criteria (A and B) were fulfilled. First (A), isolates commonly regarded as contaminants (coagulase-negative staphylococci, *Corynebacterium* spp., *Macrococcus* spp., *Micrococcus* spp., and *Facklamia* spp.), as described by previous studies and guidelines, were considered to be potentially contaminant if they grew in three or fewer of the six bottles. Second (B), the potential contaminants had to show no growth in any other relevant microbiological sample (urine, skin/soft tissue, lower respiratory tract, cerebrospinal fluid, pleural/ascitic drainage) within ±5 days of BC sampling.

### Endpoints

The main endpoint of the study was detection of relevant growth in BC. Secondary endpoint was contamination growth.

### Statistical Analysis

Based on three recently published studies (Edman-Waller et al., 2016; Rosenqvist et al., 2017; Seymour et al., 2017), we estimated that approximately 30% of cases with clinically suspected sepsis would be BC positive with both MSS and SSS. A non-inferiority margin of 5% was chosen as an acceptable limit for the 95% confidence interval (CI) of the absolute difference between the proportions of positives of the two study methods. The 95% CI of this difference was calculated using the Wald test. To obtain a power of 80% with an α level of 0.05, 520 episodes with both SSS and MSS performed were required.

Frequencies and percentages were used to summarize categorical variables, while means and standard deviations, together with medians and interquartile ranges, were used to summarize numerical variables. McNemar’s χ^2^-test was used to compare proportions between MSS and SSS.

### Ethical Permit

Ethical approval for the study was granted by the Regional Ethical Committee in Stockholm (reference number 2017/1358-31). During the study period, six BC bottles were collected in sepsis alert patients as a clinical routine. Thus, the ethics committee approved that written informed consent was not needed for inclusion in the study.

## Results

**FIGURE 1:**
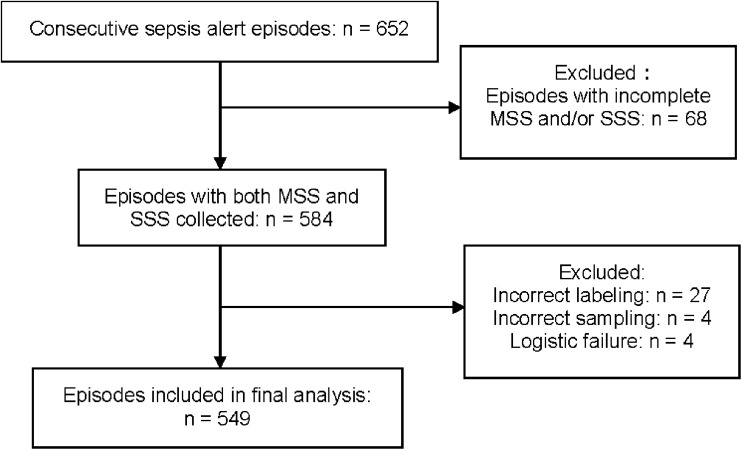
Flowchart of the study population. MSS, multi-sampling strategy; SSS, single-sampling strategy. Episodes with fewer than six blood culture (BC) bottles were considered as having MSS and/or SSS incomplete.

In total, 652 episodes triggered the sepsis alert system during the study period. Figure 1 depicts the study flowchart and reason for exclusion. After exclusion, 549 episodes from 514 unique patients were included in the final analysis. In total, 1,647 BC sets (3,294 BC bottles) were collected, each consisting of one BacT/Alert FA Plus and one FN Plus bottle. Clinical characteristics of included episodes are presented in Table 1. Of the 31 patients who had more than one episode included in the study, 27 patients had two episodes and four had three.

**TABLE 1:** Clinical characteristics of the episodes (*N* = 549 episodes).

**Characteristic**	**Value**
Male–n (%)	338 (61.6)
Age–years*	69.5 ± 16.7
**Comorbidities, n (%)**	
Congestive heart failure	95 (17.3)
Diabetes mellitus	166 (30.2)
Ischemic heart disease	71 (12.9)
Peripheral vascular disease	51 (9.3)
Cerebrovascular disease	107 (19.5)
Malignancy	130 (23.7)
Chronic kidney failure (glomerular filtration rate < 60 ml/min)	135 (24.6)
Chronic liver failure	17 (3.1)
Chronic pulmonary disease	89 (16.2)
At least one of above listed comorbidities	265 (48.3)
**Subgroup category after chart review n (%)**	
Sepsis according to sepsis-3**	387 (70.5)
Infection without sepsis**	81 (14.8)
No infection	81 (14.8)
**Source of infection, n (%)**^†^	
Respiratory tract^†⁣†^	174 (37.2)
Urinary tract	102 (21.8)
Abdominal	39 (8.3)
Soft tissue/skin/skeletal/joint	37 (7.9)
Central nervous system	4 (0.9)
Endocarditis	6 (1.3)
Other/unknown	114 (24.4)
**Disease severity/outcomes**	
SOFA score at admission^‡^	3 (2–5)
Admission to intensive care unit during hospital stay, n (%)	46 (8.4)
28-day mortality, n (%)	73 (13.3)

* Denotes mean ± standard deviation. ** Sepsis was determined as present if there was an increase in Sequential Organ failure Assessment (SOFA) score by 2 compared to baseline, as well as evidence of infection. Infection in this study was defined as present if the patient was administered intravenous antibiotic therapy within 48 h from admission and during at least 4 days. *^†^Source of infection was analyzed only for patients with infection (n = 468). Two or more sources of infection were found in nine episodes, so total percentage exceeds 100%. ^†⁣†^Includes lower and upper respiratory tract infections. ^‡^Denotes median (interquartile range).*

Growth in BC was noted in 209/549 (38.1%) episodes (in 200 unique patients) with the six study BC bottles. Supplementary Table S2A lists the detected microorganisms. Pathogenic microorganisms were found in 170 episodes (31.0%), of which 11 concomitantly had contaminant growth. A total of 39 episodes (7.1%) had only contaminant growth. Among 170 episodes with relevant growth, monomicrobial growth was detected in 140 cases (82.4%) and polymicrobial growth in 30 (17.6%). Of the polymicrobial episodes, 21 episodes had two different pathogenic microorganisms detected, and nine episodes had three different pathogenic microorganisms detected.

### Comparison of Sampling Strategies

#### Detection of Relevant Growth

**FIGURE 2:**
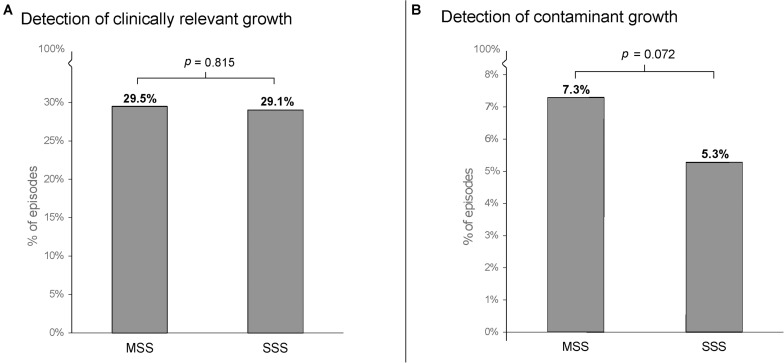
Detection rates of episodes with relevant growth **(A)** and contaminant growth **(B)** in 549 sepsis alert episodes. BC, blood culture; MSS, multi-sampling strategy; SSS, single-sampling strategy. *P*-values denote comparison using McNemar’s χ^2^-test.

Among 549 study patients, relevant growth was noted in 162 episodes (29.5%) episodes by MSS and in 160 episodes (29.1%) by SSS (Figure 2A). The absolute difference in proportion was 0.36% with 95% CI of –1.33 to 2.06%, not exceeding the non-inferiority margin of 5% (Supplementary Figure S1). Thus, SSS was found to be non-inferior to MSS for detection of relevant growth in BC.

If detection of all pathogenic microorganisms in the six study BC bottles would have been required for a positive result, MSS and SSS would have been positive in 160 episodes (29.1%) and 150 episodes (27.3%), respectively. The difference in proportion would then have been 1.8% with a 95% CI of –0.31 to 3.95%, not exceeding the non-inferiority margin of the study.

MSS and SSS performed similarly in monomicrobial growth and could detect microorganisms in 132 (24.0%) and 130 (23.7%) episodes, respectively (NS).

When polymicrobial samples were analyzed, both MSS and SSS detected growth in all 30 episodes. When detection of all microorganisms in polymicrobial episodes was considered, MSS [28/30 (93%)] detected a higher rate of all microorganisms compared to SSS [22/30 (73%)], although not reaching statistical significance (*p* = 0.08) (Tables 2, 3).

**TABLE 2:** Detection of microorganisms by MSS and SSS* (*N* = 549 episodes).

**Episode type**	**MSS**	**SSS**
**Episodes with clinically relevant growth, n (%)**	**162 (29.5)**	**160 (29.1)**
Monomicrobial episodes	132 (24.0)	130 (23.7)
Polymicrobial episodes**	30 (5.5)	30 (5.5)
Episodes including G+ isolates	72 (13.1)	72 (13.1)
Episodes including G- isolates	103 (18.8)	96 (17.5)
Episodes including fungal isolates	1 (0.2)	0
Episodes including anaerobic isolates	11 (2.0)	10 (1.8)
**Episodes with contaminant growth, n (%)**	**40 (7.3)**	**29 (5.3)**
Only contaminant growth	34 (6.2)	21 (3.8)
Both contaminant and clinically relevant growth	6 (1.1)	8 (1.5)

* MSS, multi-sampling strategy; SSS, single-sampling strategy; BC, blood culture. ** Polymicrobial episode is defined as an episode with the occurrence of more than one clinically relevant isolate.

**TABLE 3:** Discordant results between MSS and SSS* in episodes with polymicrobial growth.

**Site of infection**	**Isolates detected by both methods**	**Isolates detected by MSS only**	**Isolates detected by SSS only**
Abdomen	*Escherichia coli*	*Enterococcus faecium*	*–*
Abdomen	*Klebsiella pneumoniae, Enterococcus faecalis*	*Stenotrophomonas maltophilia*	*–*
Abdomen	*E. coli*		*E. faecium*
Dental	*Streptococcus anginosus*	*Parvimonas micra*	*–*
Lower respiratory tract	*E. coli*	*–*	*K. pneumoniae*
Soft tissue	*Streptococcus pneumoniae*	*E. coli*	*–*
Soft tissue	*Streptococcus pyogenes*	*Staphylococcus aureus*	*–*
Urinary tract	*E. coli, S. aureus*	*E. faecalis*	*–*
Unknown	*Citrobacter freundii, S. aureus*	*E. faecalis*	*–*
Unknown	*Helcococcus* spp.	*Globicatella* spp*., Alcaligenes faecalis*	*–*
Unknown	*E. coli*	*Candida parapsilosis, Lactobacillus* spp.	*–*
Unknown	*Actinomyces* spp.	*Streptococcus mitis*	*–*

Site of infection and isolated microorganisms are presented. * MSS, multi-sampling strategy; SSS, single-sampling strategy; Spp., species.

Monomicrobial episodes with discordant findings are described in Supplementary Table S1. There was a similar proportion of Gram-positive and Gram-negative episodes detected by the methods.

#### Contaminant Growth

Figure 2B shows the detection of contaminant growth with the two sampling methods studied. SSS (5.3%) tended to yield a lower rate of contaminants than MSS (7.3%), although not to statistical significance (*p* = 0.07). Supplementary Table S2b shows the contaminants detected by MSS and SSS.

### Growth in Individual Blood Culture Bottles

Growth of any microorganism was observed in 784/3,294 (23.8%) bottles. The overall contamination rate in BC bottles was 75/3,294 (2.28%). Growth in the individual BC bottles (BC1, BC2, BC3, BC4, BC5, BC6) was evenly distributed (Table 4).

**TABLE 4:** Distribution of growth in individual blood culture bottles (*N* = 549 episodes).

	**MSS and SSS common bottles**		**SSS**		**MSS**	
	**BC bottle 1**	**BC bottle 2**	**BC bottle 3**	**BC bottle 4**	**BC bottle 5**	**BC bottle 6**
Clinically relevant growth, n (%)	140 (25.5)	129 (23.5)	131 (23.9)	116 (21.1)	139 (25.3)	129 (23.5)
Contaminant growth, n (%)	14 (2.6)	10 (1.8)	11 (2.0)	7 (1.3)	16 (2.9)	17 (3.1)

**MSS, multi-sampling strategy; SSS, single-sampling strategy; BC, blood culture.**

## Discussion

Appropriate detection of microorganisms and low rate of contaminants are two major goals for BC diagnostics. Therefore, the sampling strategy is essential to optimize the performance of BC. In the present study, a well-defined patient cohort with clinically suspected sepsis was used to evaluate if SSS is non-inferior to MSS for detection of relevant growth in BC.

For SSS, the main concern has been that diagnostic performance may be lower than that of MSS (Lamy et al., 2016). The present study shows that SSS is non-inferior to MSS regarding detection of pathogenic microorganisms in BC. These results are the first to demonstrate this non-inferiority and confirm previous theoretical assumptions based on a statistical model by Lamy et al. (2002) and empirical data published more recently by Dargere et al. (2014). Non-inferiority of SSS compared to MSS combined with advantages of SSS in terms of less harm to the patient and less labor intensity for the staff supports the use of SSS as the routine method for BC in emergency departments.

Contamination is a major problem with BC diagnostics, and a reduction of contaminants is one of the proposed benefits of using SSS (Lamy et al., 2016). In the present study, the overall contamination rate per BC bottle was less than 3%, which is the upper limit of the accepted contamination rate (Dargere et al., 2018). SSS tended to yield a lower rate of contaminants than MSS, although not to statistical significance (*p* = 0.07). In the current study, a wide range of different possible contaminants were detected with the exception of *Cutibacterium acnes*. The underlying reason for not detection of *Cutibacterium acnes* is not known but can probably be related to the BC system used in the present study. The present results on contamination rates are in line with previously published data (Dargere et al., 2014).

The performance of BC in the detection of polymicrobial growth has not been studied extensively. Around 5–10% of positive BCs have previously been reported to be polymicrobial (Arendrup et al., 1996; Lee et al., 2007; Lin et al., 2010; Dargere et al., 2014). Here, polymicrobial episodes represented 17.6% of all relevant growth. The reason for this high frequency may be a higher rate of comorbid illnesses and a higher proportion of true sepsis in the present cohort. In the present study, there was no difference between MSS and SSS in detecting polymicrobial growth. However, when analyzing the individual detection of all microorganisms in polymicrobial episodes, MSS detected all microorganisms more often than in SSS. However, statistical comparison for non-inferiority could not be performed due to small numbers of polymicrobial samples. Further studies including a large number of cases with polymicrobial sepsis are warranted.

This study has some limitations. First, the exclusion of almost 100 sepsis alert episodes due to insufficient number of BC bottles may have influenced the mix of etiologies and/or severity of the studied patient cohort. However, such influence would most likely not affect the study results substantially since the patients were their own controls. Second, the sample size was too small to evaluate if SSS would be superior to MSS regarding contamination rate. However, the results of the present study could be useful for sample size calculation of such a study. Third, the BC bottle volumes were not measured because of logistical difficulties, and thus it is unclear if the blood volumes of BC bottles 5 and 6 and BC bottles 3 and 4 were similar in individual patients. However, the similar rates of growth observed in the parallel bottles suggest that the blood volumes did not differ significantly among individual bottles.

The present study has several strengths. The well-defined sepsis alert system used in the present study resulted in a high rate of positive BCs and low contamination rates, two parameters that are decisive in an analysis of the performance of sampling methods. Both sampling methods (MSS and SSS) were performed simultaneously during the initial management in the Emergency Department, having the patients as their own controls. The samples were collected meticulously with pre-labeled BC bottles and clear instructions, leaving little room for ambiguity. In addition, the present approach used here minimized the risk for change in preconditions, most importantly initiation of antibiotic therapy.

## Conclusion

The present study shows that SSS is non-inferior to MSS in the detection of pathogenic microorganisms in BC. This fact combined with advantages of SSS in terms of less harm to the patient and less labor intensity for the staff supports the use of SSS as a routine method for BC in emergency departments. Clinical studies with a larger sample size comparing the two sampling methods in the detection of contaminants and all microorganisms with polymicrobial growth are warranted.

## Data Availability Statement

The raw data supporting the conclusions of this article will be made available by the authors, without undue reservation, to any qualified researcher.

## Ethics Statement

The studies involving human participants were reviewed and approved by the Regional Ethical Committee in Stockholm (reference number 2017/1358-31). During the study period, 6 BC bottles were collected in sepsis alert patients as a clinical routine. Thus, the ethics committee approved that written informed consent was not needed for inclusion in the study. Written informed consent for participation was not required for this study in accordance with the national legislation and the institutional requirements.

## Author Contributions

DY, KS, and VÖ were involved in the study design, sample collection, analysis of the data, and writing the manuscript. AL was involved in analysis of the data and writing the manuscript. ÅP and CU were involved in the study design and sample collection. CH and JS-C was involved in the study design. AS was involved in sample collection. All authors contributed to the article and approved the submitted version.

## Conflict of Interest

The authors declare that the research was conducted in the absence of any commercial or financial relationships that could be construed as a potential conflict of interest.

## Supplementary Material

FIGURE S1 Observed absolute difference and 95% confidence interval (CI) between detection rates for MSS and SSS. MSS, Multi-sampling strategy; SSS, Single-sampling strategy. The diamond represents the observed absolute difference and the horizontal line represents the 95% CI, calculated using the Wald test. The non-inferiority margin Δ is represented by the dotted line.

TABLE S1 Discordant results between MSS and SSS* in episodes with monomicrobial growth. Site of infection and isolated microorganisms are presented. *MSS: multi-sampling strategy. SSS: single-sampling strategy. Spp.: species.

TABLE S2 **(a)** Rank order of clinically relevant isolates *Serratia marcescens, Proteus mirabilis, Pantoea species, Citrobacter freundii, Moraxella catharralis, Aeromonas caviae, Hafnia alvei, Alcaligenes faecalis, Acinetobacter species, Campylobacter species, Stenotrophomonas maltophilia, Proteus vulgaris, Pseudomonas species, Haemophilus influenzae, Enterobacter aerogenes. ** Streptococcus agalactiae, Group A beta-hemolytic streptococcus, Coagulase negative staphylococcus, Streptococcus mitis, Parvimonas micra, Group B beta-hemolytic streptococcus, Enterococcus species, Globicatella species, Helcococcus species, Streptococcus anginosus, Streptococus anginosus, Lactobacillus species, Group G beta-hemolytic streptococcus, Enterococcus casseliflavus, Actinomyces species, Macrococcus species, Micrococcus luteus *** Prevotella species, Clostridium perfringens, Peptoniphilus species, Peptostreptococcus anaerobius, one isolate that could not be identified further **(b)** Rank order of contaminant isolates.
