# Supplementary figures and images for: Single-Sampling Strategy vs. Multi-Sampling Strategy for Blood Cultures in Sepsis: A Prospective Non-inferiority Study
Source: Front Microbiol. 2020 Jul 23;11:1639. doi: 10.3389/fmicb.2020.01639 (PMC7390949; doi:10.3389/fmicb.2020.01639)

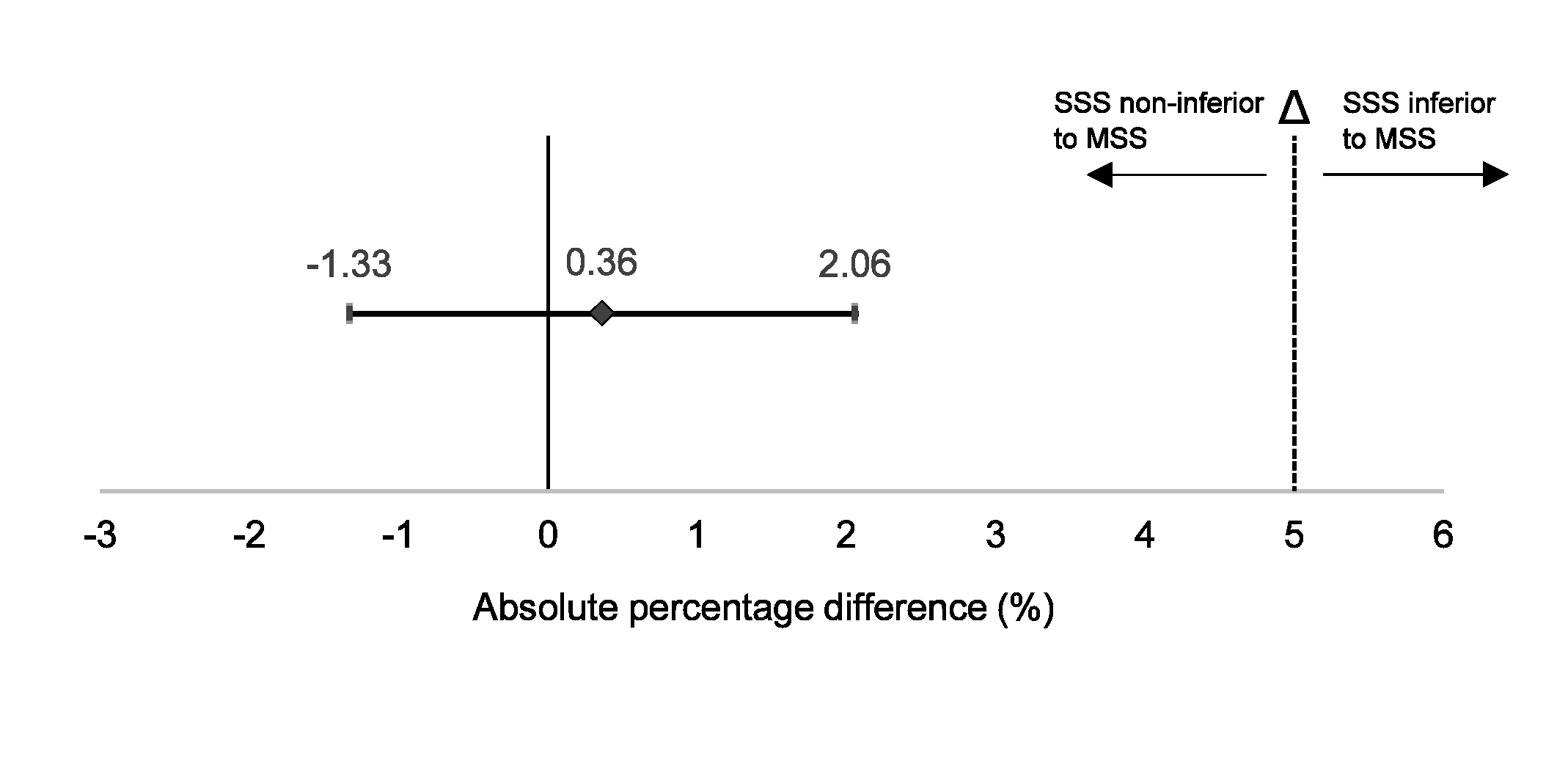

Supplement: FIGURE S1 — Observed absolute difference and 95% confidence interval (CI) between detection rates for MSS and SSS. MSS, Multi-sampling strategy; SSS, Single-sampling strategy. The diamond represents the observed absolute difference and the horizontal line represents the 95% CI, calculated using the Wald test. The non-inferiority margin Δ is represented by the dotted line. [file Image_1.TIFF]
