# Supplementary material for: Single-Sampling Strategy vs. Multi-Sampling Strategy for Blood Cultures in Sepsis: A Prospective Non-inferiority Study
Source: Front Microbiol. 2020 Jul 23;11:1639. doi: 10.3389/fmicb.2020.01639 (PMC7390949; doi:10.3389/fmicb.2020.01639)
Supplement: TABLE S1 — Discordant results between MSS and SSS* in episodes with monomicrobial growth. Site of infection and isolated microorganisms are presented. *MSS: multi-sampling strategy. SSS: single-sampling strategy. Spp.: species. [file Data_Sheet_1.PDF]

Supplementary Table 1: Discordant results between MSS and SSS* in episodes with monomicrobial growth. Site of infection and isolated microorganisms are presented.

Site of infection	Isolates detected by MSS	Isolates detected by SSS
Abdomen	-	***Enterococcus spp.***
Abdomen	***Escherichia coli***	-
Catheter related bloodstream infection	-	***Staphylococcus epidermidis***
Lower respiratory tract	-	***Streptococcus pneumoniae***
Lower respiratory tract	-	***S. aureus***
Lower respiratory tract	***S. aureus***	-
Urinary tract	-	***Citrobacter freundii***
Urinary tract	***Pseudomonas aeruginosa***	-
Urinary tract	-	***E. coli***
Urinary tract	***E. coli***	
Unknown	-	***S. epidermidis***
Unknown	***P. aeruginosa***	-
Unknown	***Pantoea* spp.**	-
Unknown	***Pantoea* spp.**	-
Unknown	***P. aeruginosa***	-
Unknown	-	***Streptococcus agalactiae***
Unknown	***S. aureus***	-
Unknown	***P. aeruginosa***	-
Unknown	-	***Acinetobacter species***

*MSS: multi-sampling strategy. SSS: single-sampling strategy. Spp.: species.

Supplementary Table 2a: Rank order of clinically relevant isolates

**Gram negative bacteria**	**n**	**%**
Escherichia coli	55	26.1%
Klebsiella pneumoniae	15	7.1%
Klebsiella oxytoca	9	4.3%
Pseudomonas aeruginosa	8	3.8%
Enterobacter cloacae	4	1.9%
Other gram negative bacteria*	21	10.0%
**Total gram negative bacteria:**	**112**	**53.1%**
**Gram positive bacteria**	**n**	**%**
Staphylococcus aureus	22	10.4%
Staphylococcus epidermidis	12	5.7%
Enterococcus faecalis	10	4.7%
Enterococcus faecium	7	3.3%
Streptococcus pneumoniae	6	2.8%
Other gram positive bacteria**	28	16.1%
**Total gram positive bacteria**	**85**	**40.3%**
**Anaerobic bacteria**	**n**	**%**
Bacteroides fragilis	3	1.4%
Parvimonas micra	2	0.9%
Actinotignum schaalii	1	0.5%
Helcococcus spp	1	0.5%
Eggerthella lenta	1	0.5%
Other anaerobic bacteria***	5	2.4%
**Total anaerobic bacteria**	**13**	**6.1%**
**Fungi**	**n**	**%**
Candida parapsilosis	1	0.5%
**Total number of clinically relevant isolates:**	**211**	**100%**

Serratia marcescens, Proteus mirabilis, Pantoea* species, *Citrobacter freundii, Moraxella catharralis, Aeromonas caviae, Hafnia alvei, Alcaligenes faecalis, Acinetobacter* species, *Campylobacter* species, *Stenotrophomonas maltophilia, Proteus vulgaris, Pseudomonas* species, *Haemophilus influenzae, Enterobacter aerogenes. ** Streptococcus agalactiae,* Group A beta-hemolytic streptococcus, Coagulase negative staphylococcus, *Streptococcus mitis, Parvimonas micra,* Group B beta-hemolytic streptococcus, *Enterococcus* species, *Globicatella* species, *Helcococcus* species, *Streptococcus anginosus, Streptocous anginosus, Lactobacillus* species, Group G beta-hemolytic streptococcus, *Enterococcus casseliflavus, Actinomyces* species, *Macrococcus* species, *Micrococcus luteus * Prevotella* species, *Clostridium perfringens, Peptoniphilus* species, *Peptostreptococcus anaerobius,* one isolate that could not be identified further

Supplementary Table 2b: Rank order of contaminant isolates.

**Contaminant isolates**	**n**	**%**
Coagulase negative staphylococcus	29	51.8%
Staphylococcus epidermidis	20	35.7%
Corynebacterium spp.	4	7.1%
Macrococcus spp.	1	1.8%
Facklamia spp.	1	1.8%
Micrococcus luteus	1	1.8%
**Total number of contaminant isolates**	**56**	**100%**
